# Classification of Frozen Corn Seeds Using Hyperspectral VIS/NIR Reflectance Imaging

**DOI:** 10.3390/molecules24010149

**Published:** 2019-01-02

**Authors:** Jun Zhang, Limin Dai, Fang Cheng

**Affiliations:** College of Biosystems Engineering and Food Science, Zhejiang University, Hangzhou 310058, China; 11613006@zju.edu.cn (J.Z.); lmdai@zju.edu.cn (L.D.)

**Keywords:** VIS/NIR hyperspectral imaging, corn seed, classification, freeze-damaged, image processing, imaging visualization

## Abstract

A VIS/NIR hyperspectral imaging system was used to classify three different degrees of freeze-damage in corn seeds. Using image processing methods, the hyperspectral image of the corn seed embryo was obtained first. To find a relatively better method for later imaging visualization, four different pretreatment methods (no pretreatment, multiplicative scatter correction (MSC), standard normal variation (SNV) and 5 points and 3 times smoothing (5-3 smoothing)), four wavelength selection algorithms (successive projection algorithm (SPA), principal component analysis (PCA), X-loading and full-band method) and three different classification modeling methods (partial least squares-discriminant analysis (PLS-DA), K-nearest neighbor (KNN) and support vector machine (SVM)) were applied to make a comparison. Next, the visualization images according to a mean spectrum to mean spectrum (M2M) and a mean spectrum to pixel spectrum (M2P) were compared in order to better represent the freeze damage to the seed embryos. It was concluded that the 5-3 smoothing method and SPA wavelength selection method applied to the modeling can improve the signal-to-noise ratio, classification accuracy of the model (more than 90%). The final classification results of the method M2P were better than the method M2M, which had fewer numbers of misclassified corn seed samples and the samples could be visualized well.

## 1. Introduction

Corn (*Zea mays* L.), one of the world’s three major food crops, is currently one of the most grown food crops in several parts of the world [1]. As the world’s second-largest corn producing and consuming country, China has most of its corn growing areas located in the north. In these regions, the corn seed is often damaged due to low temperatures and high seed moisture content before harvest or dehydration, which is an agricultural disaster.

Seed embryo is the most important part of the seed which contains a large number of nutrients. If damage takes place in this part, it must have great impact on subsequent growth. After suffering from low-temperature freeze damage, the seed quality declines, and it is easy for mildew to grow when the seeds are stored at later stage. The internal components of the seed will change, which results in a great impact on the subsequent germination, root growth and development. To investigate the vigor change in seed and how it changes, the International Seed Testing Association (ISTA) recommended two kinds of seed viability measurement methods in 1995 [2], the electrical conductivity test could be conveniently conducted due to its simplicity and low cost [3,4].

Therefore, a key factor in current study is how to quickly and accurately determine the characteristic changes in the freeze-damage seeds and identify the freeze condition (especially, the slightly freeze-damaged seeds), which will provide guidance for the seed agricultural production. In particular, it is of more specific significance to study the frost damage status of the embryo.

In recent studies, through the use of near-infrared spectroscopy technology, the vitality [5], internal essential constituents such as lipids [6], starches [7,8] and the toxin-infected pests [9,10,11] to corn seed batches have been studied, all of which have a rapid non-destructive advantage, but this technology only processes the corn seed in batches, and it is hard to determine the characteristics of individual corn seeds.

At present, the application of hyperspectral imaging technology in non-destructive testing of agricultural products has become more extensive. As a technology that combines the advantages of traditional image technology and spectral technology, it can obtain the spectral information of every pixel on the collected image, which can be used to effectively analyze the chemical composition index of each part of the seed and avoid the instability of experimental results. Another advantage of this technology is that it can test the seeds individually. Many research studies such as those on water content [12], hardness [13,14], internal component testing [15], variety classification [16,17,18,19], vitality [1,20], different storage periods [21,22,23], fungi and toxin detection [24,25,26,27,28,29,30,31,32] have been reported. Huang et al. (2015) used hyperspectral imaging techniques to predict the consistency of seed moisture content with correlation coefficient of prediction set of 0.848 [33]; Williams et al. (2016) used NIR hyperspectral imaging to classify maize kernels into three hardness categories, where pixel-wise and object-wise methods were compared and they had similar results [34]; Zhao et al. (2018) used hyperspectral imaging techniques to study a total of 12,900 maize seeds of 3 different varieties, first to determine the optimal calibration set of each variety, and then the performance of the back propagation neutral network and support vector machine models were compared to obtain the best model, the overall results indicated that hyperspectral imaging was a potential technique for varietal classification of maize seeds [35].

According to the current research, no study has been conducted on the freeze damage of corn seed, although there are some studies using hyperspectral imaging techniques to study frozen grown crops [36,37,38,39] or fruit [40], but these studies could not be used for the freeze damage identification of the seeds due to different technical methods and objectives.

To summarize, the objectives of this study were to: (1) conduct an electrical conductivity test on corn seeds to consider if the seed is damaged or not; (2) obtain the corn seed embryo hyperspectral image, and assess the potential of applying hyperspectral imaging technology for the classification of different degrees of freeze damage to the corn seeds; (3) evaluate the models established by different spectral pretreatment methods, wavelength selection methods and modeling methods, and then compare and identify the optimal model among them; (4) visualize the classification results of two different methods (M2M and M2P), and to identify the optimal method.

## 2. Results and Discussions

### 2.1. Results of Conductivity Test

**Figure 1:**
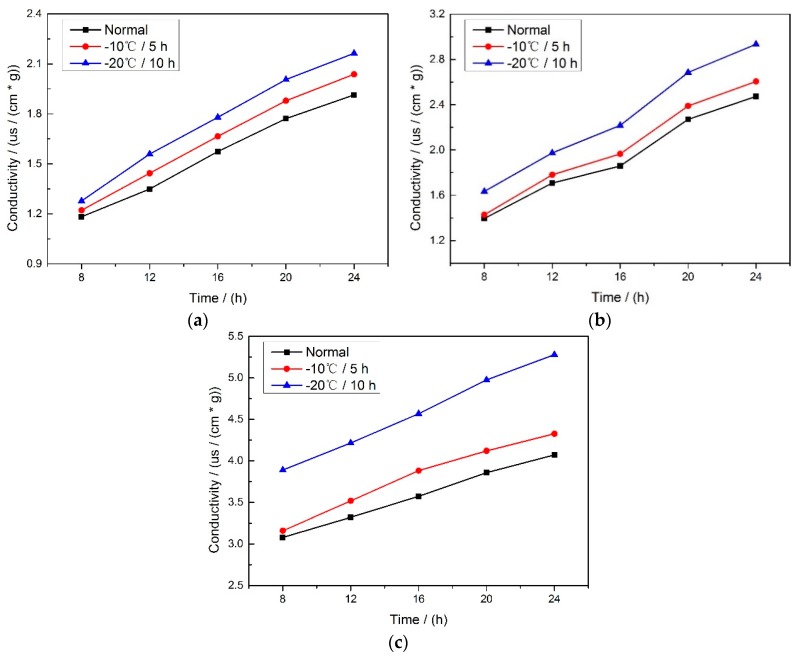
The conductivity of (**a**) Haoyu21, (**b**) Haihe78 and (**c**) Jindan10 soaking for 24 h.

Figure 1 shows the results of the conductivity of three varieties of corn seeds after soaking for 24 h, it can be found that the highest conductivity is at the frost condition of −20 °C, for 10 h, the second is at −10 °C, for 5 h while the lowest is at the normal condition. It indicates that during the freeze-damage process, the membrane integrity of the corn seed deteriorated, and then the leakage of the cell contents was serious after the seeds absorbing water, thus a higher conductivity was obtained.

### 2.2. The Analysis of Spectral Features

**Figure 2:**
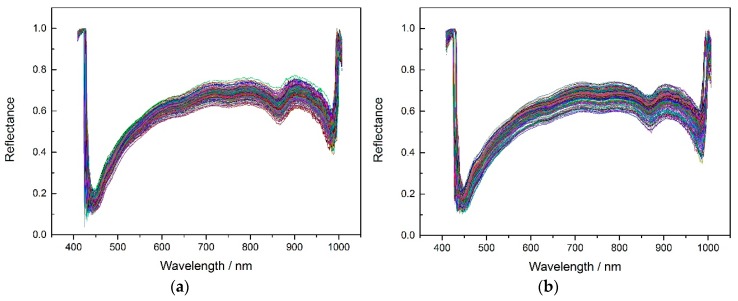

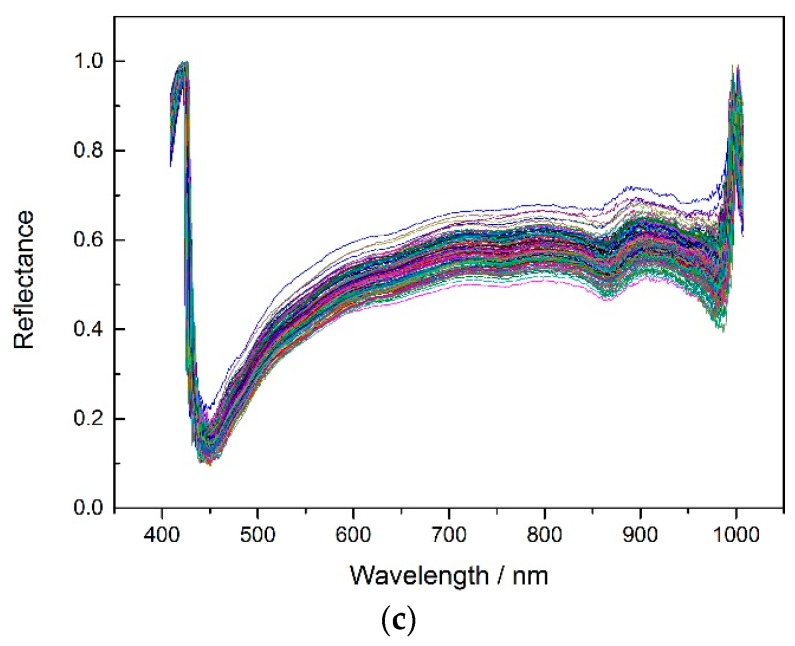
The average spectrums of (**a**) Haoyu21, (**b**) Haihe78 and (**c**) Jindan10.

According to the experiment description, Figure 2 shows the average spectrums of three different corn seeds varieties.

In order to get better classification results, the noise wavelengths (before 444.23 nm and after 985.37 nm) were excluded and the remaining 430 wavelengths were used for later modeling. The SPA, PCA and X-loading methods were applied to select feature wavelengths after applying the pretreatment methods (no pretreatment, MSC, SNV and 5-3 smoothing) to the spectrums. The feature wavelength results for the three varieties are shown in appendixes Table A1, Table A2 and Table A3.

**Figure 3:**
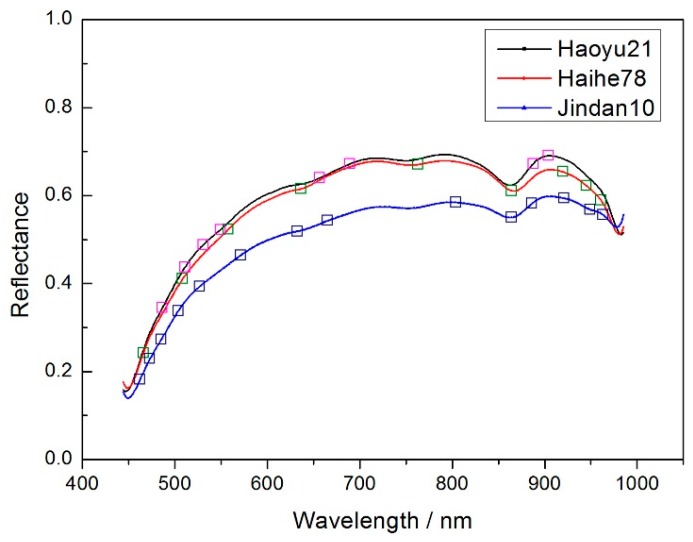
The description of the selected wavelengths by 5-3 smoothing pretreatment and the SPA method for the three varieties.

From Table A1, Table A2 and Table A3, the original input of 430 wavelengths was dramatically reduced to several or no more than 20 inputs, thus the calculating time was greatly shortened. To take the selected wavelengths in the condition of 5-3 smoothing pretreatment method and SPA method as an example (Figure 3). Most of the selected wavelengths were in the range of 450–700 nm, they are possibly related to the change in Chlorophyll, β-carotene or other components related to the embryo [41]. In the range of 850–950 nm, it is mainly related to the 3rd overtune vibrations of the hydrocarbon C-H bond [42].

### 2.3. The Results of Established Classification Models

**Figure 4:**
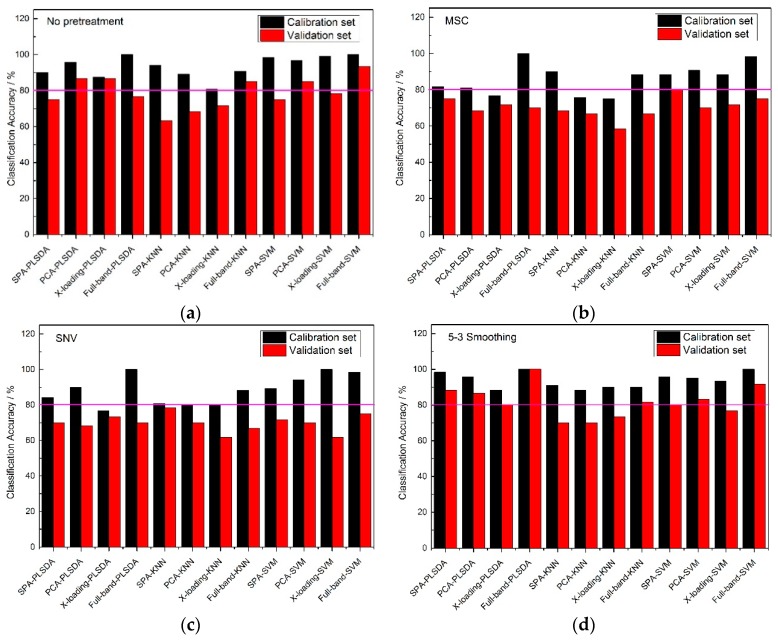
The classification accuracy results of Haoyu21 with (**a**) no pretreatment method, (**b**) the MSC pretreatment method, (**c**) the SNV pretreatment method and (**d**) the 5-3 smoothing pretreatment method.

**Figure 5:**
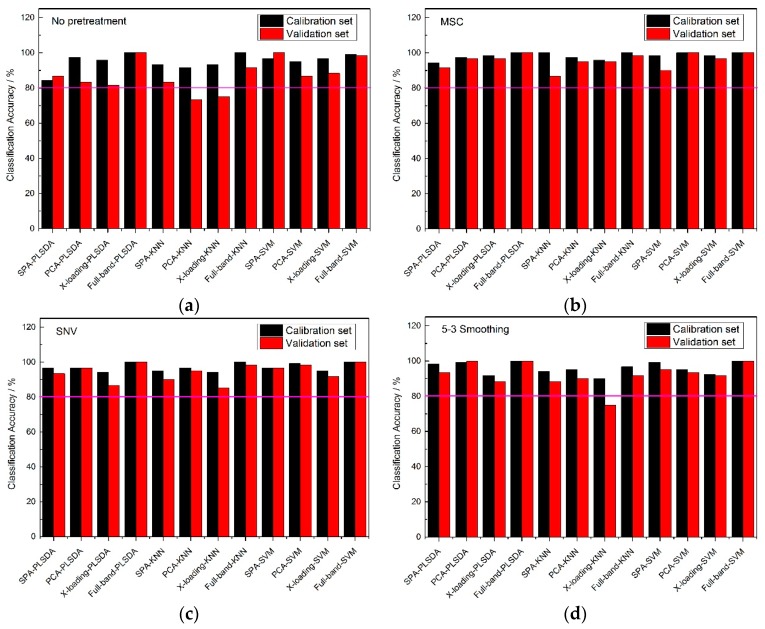
The classification accuracy results of Haihe78 with (**a**) no pretreatment method, (**b**) the MSC pretreatment method, (**c**) the SNV pretreatment method and (**d**) the 5-3 smoothing pretreatment method.

**Figure 6:**
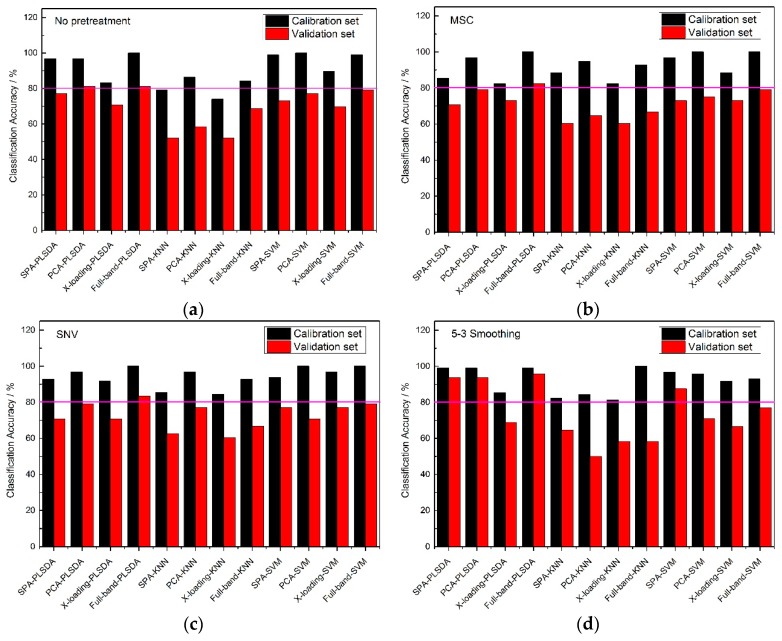
The classification accuracy results of Jindan10 with (**a**) no pretreatment method, (**b**) the MSC pretreatment method, (**c**) the SNV pretreatment method and (**d**) the 5-3 smoothing pretreatment method.

When the feature wavelengths were selected, the classification models were established. The accuracy results of the three varieties of corn seed are shown as Figure 4, Figure 5 and Figure 6.

From Figure 4, an 80% pink line for both calibration sets and validation sets was firstly set. At full-band treatment, the classification accuracy results of 5-3 smoothing and no pretreatment method were better than those of the MSC and SNV pretreatment methods, because the results of the validation sets with the MSC and SNC pretreatment methods were all lower than 80%. With the same pretreatment method, the classification accuracies of the full-band, SPA, PCA and X-loading methods were sorted: The classification accuracies for the full-band method were higher than those of SPA, and those of SPA were higher than those of the PCA and X-loading methods. With the same pretreatment method and the same wavelength selection algorithm, both the PLS-DA and SVM modeling methods had higher accuracy results than the KNN method. The >80% classification accuracy results for the calibration set and validation set with the KNN method only appeared in no pretreatment and 5-3 smoothing pretreatment method.

By counting the number of >80% classification accuracy results, the 5-3 smoothing and no pretreatment method had similar classification result, and in the meantime, the PLS-DA and SVM modeling methods had similar classification result.

From Figure 5, the classification accuracy results of the no pretreatment and three pretreatment methods at full band, were very high, and most of them could reach an accuracy of 100%, which perhaps shown that a wonderful classification model could be established on the premise of it containing all the reflectance spectral information of the samples. Though there were good accuracy results among each pretreatment method for full-band spectrums, irrelevant information for the sample is still existed, so it was necessary to find several wavelengths to represent the 430 wavelengths to reduce the calculation time. With the same pretreatment methods, the classification accuracies of the full-band, SPA, PCA and X-loading methods were sorted: the classification accuracies for the full-band method were higher than those of the SPA, and those for SPA and PCA methods were higher than those of the X-loading method. In some respects, it could be found that many of the classification accuracies for PCA were slightly higher than those for SPA, but the number of > 80% classification accuracies for SPA was one more than that for PCA. Though the full-band method had the best classification results among the four pretreatment methods, the accuracies of SPA were also much higher, and most of the accuracies of the validation sets were almost more than 90%. So it could also be used for the classification of frozen corn seeds. Moreover, with the same pretreatment method and the same wavelength selection algorithm, the PLS-DA and SVM modeling methods had higher accuracy results than the KNN method. 

By counting the number of >80% classification accuracies, all of the pretreatment methods had similar classification result, and in the meantime, the number of >80% classification accuracy results for the calibration set and validation set of the KNN method were fewer than for the other two classification modeling methods.

From Figure 6, the 5-3 smoothing pretreatment method had a greater number of >80% classification accuracy results than the no pretreatment and the other two pretreatment methods at full band. Almost all of the calibration sets could reach an accuracy of 85% or higher, while most of the accuracies of the validation sets were lower than 80%. With the same pretreatment method, the classification accuracies of the full-band, SPA, PCA and X-loading methods were sorted: the classification accuracy results of the full-band than 80%. With the same pretreatment method and the same wavelength selection algorithm, the PLS-DA modeling method had higher accuracy results than the SVM and KNN modeling methods. There was no >80% classification accuracy result for the calibration set and validation set in KNN modeling method.

By counting the number of >80% classification accuracies, the conclusion was drawn that the classification accuracy results for the PLS-DA modeling method with the 5-3 smoothing pretreatment method had better results than any other pretreatment methods.

### 2.4. The Visualization Images of the Classification Results

A better model was found when using the 5-3 smoothing pretreatment method and the PLS-DA classification modeling method. To more clearly understand the classification results of the corn seed samples, the spectrum of each pixel in the embryo image was classified to realize the visualization of the corn embryo images.

#### 2.4.1. The Visualization Images of Haoyu21

**Figure 7:**
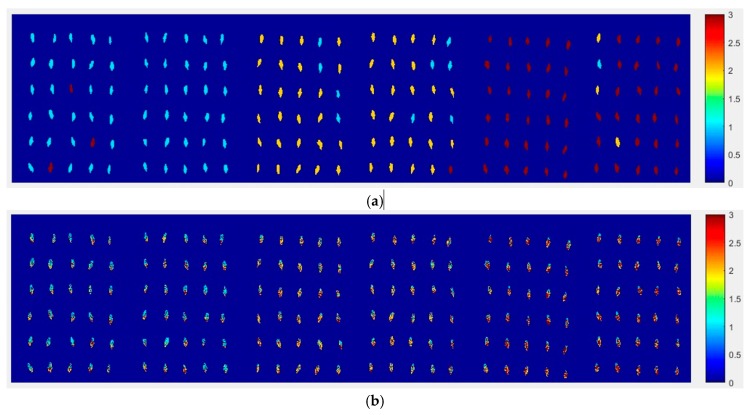
The visualization images of (**a**) method M2M and (**b**) method M2P for Haoyu21 with the SPA and PLS-DA model.

At first, the six images (the first two are of normal corn seeds, the middle two are of slightly freeze-damaged corn seeds and the last two are of severely freeze-damaged corn seeds) of three different degrees of freeze-damage in corn seed were merged, Figure 7 shows the two different classified images. Figure 7a,b, were the results images obtained by method M2M and method M2P, respectively.

From Figure 7a, the visualization image with the SPA and PLS-DA model was almost matched with the above figure results. As for Figure 7b, each pixels had a classification value and each corn seed image was obtained to form a whole image with different color gradients (from light blue to yellow and then to deep red) although not all of the pixels were the same color in one corn seed. 

**Figure 8:**
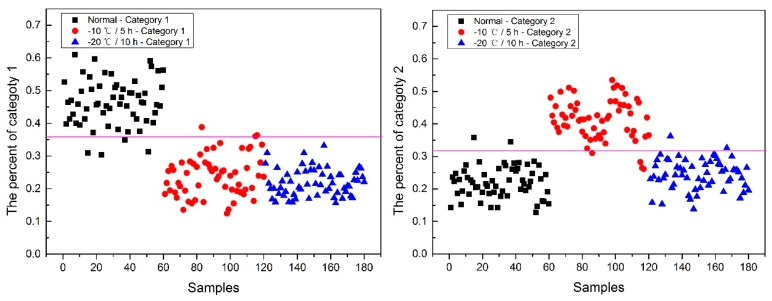

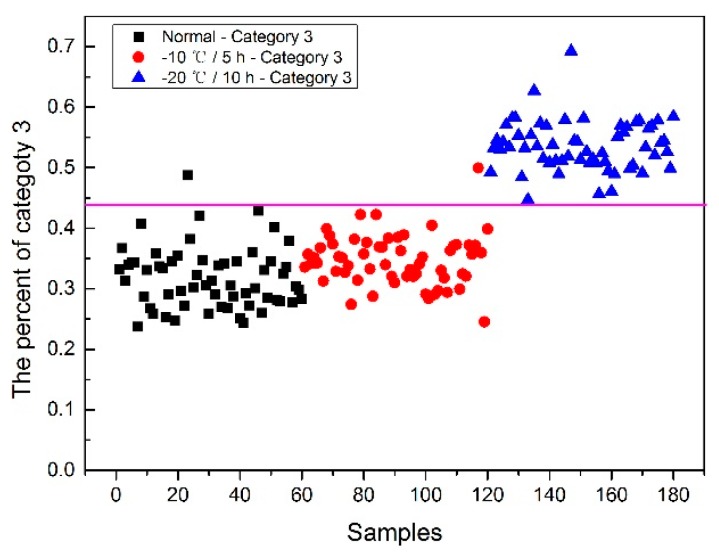
The percentage of each category of Haoyu21 with the SPA and PLS-DA model.

Now, how to identify the final category for the corn seeds was next step. The percentage of each category of each corn seed was calculated, and the results are shown in Figure 8.

From Figure 8, each type of corn seed had its own concentrated percentage distribution area. For example, the first 60 corn seed samples had a larger percentage of category 1 than those of the other corn seed samples because they were the normal corn seeds; With a threshold of 0.37, the number 83 corn seed sample was misclassified as category 1. The middle 60 corn seed samples had a larger percentage of category 2 than those of the other corn seed samples because they were the slightly freeze-damaged corn seeds; With a threshold of 0.32, the number 15, 37 and 167 corn seed samples were misclassified as category 2. The last 60 corn seed samples had a larger percentage of category 3 than those of the other corn seed samples because they were the severely freeze-damaged corn seed; With a threshold of 0.44, and all of the category 3 corn seeds were classified correctly, the number 23 and 117 corn seed samples were misclassified as category 3.

Among numbers 15, 23, 37, 83, 117 and 167, It was found that numbers 83 and 167 were classified into two categories (number 83 was classified as categories 1 and 2, and number 167 was classified as categories 2 and 3), shown in Table 1. One sample should only have one category. Thus, a method to classify them into one category need to be found. In this study, the percentages of two categories were compared, and the bigger one was the final category and the final category was obtained with the smallest value in the deep black color. In the end, the number 167 was classified correctly.

**Figure 9:**
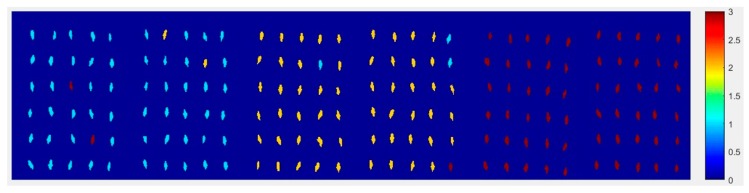
The final visualization images of Haoyu21 with the SPA and PLS-DA model with method M2P.

It was also found that the category 1 percentage of number 15, 23, 37 and 51 corn seeds were lower than 0.37, and they were not included in category 1; the category 2 percentage of number 86, 115, 116 and 117 corn seeds were lower than 0.32, and they were not included in category 2. From the above study the number 15, 23, 37 and 117 corn seeds were classified, but the 51, 86, 115 and 116 corn seeds did not have their own category, so the percentage of each category was compared shown in Table 2. After subtracting the percentage from the threshold, the final category was obtained with the smallest value in the deep black color. At last, the number 86 corn seed was classified as category 2 correctly, while the number 51 (should be category 1) was classified as category 2 and numbers 115 and 116 (should be category 2) were classified as category 1. The final classification results of method M2P are shown in Figure 9.

To compare the above results, the number of misclassified corn seed samples were counted. There were 17 corn seed samples misclassified by method M2M while eight corn seed samples were misclassified by method M2P. Meanwhile, none of the severely freeze-damaged samples were misclassified by method M2P. In some respects, it could be drawn that method M2P shown better results than method M2M.

#### 2.4.2. The Visualization Images of Haihe78 

**Figure 10:**
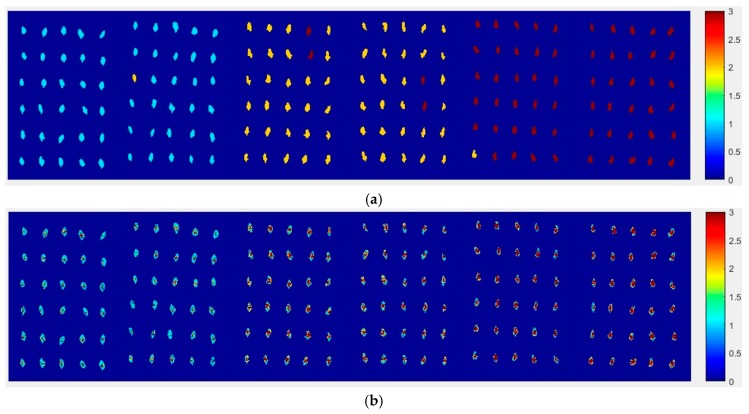
The visualization images (**a**) method M2M and (**b**) method M2P for Haihe78 with the SPA and PLS-DA model.

From Figure 10, the top visualization image (Figure 10a) with the SPA and PLS-DA model was almost matched with the above figure results. The 6 misclassified corn seed samples of Haihe78 were fewer than the 17 of Haoyu21.

**Figure 11:**
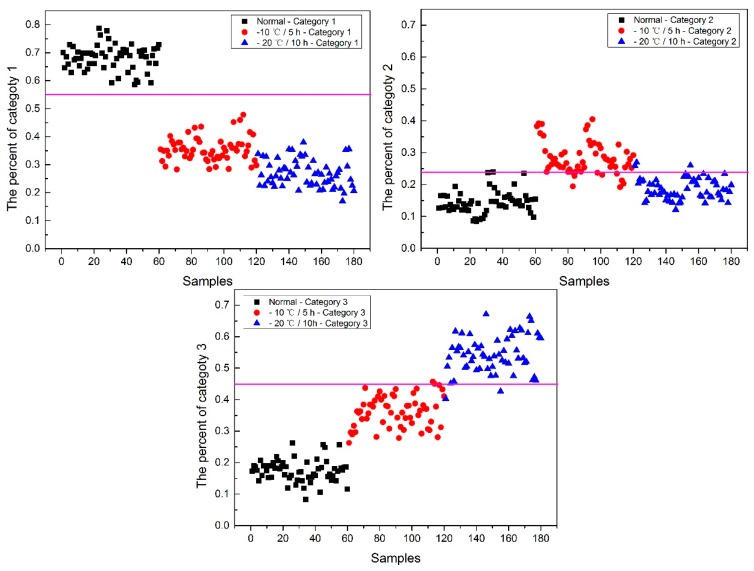
The percentage of each category of Haihe78 with the SPA and PLS-DA model.

Next, the percentage of each category of each corn seed was calculated, and the results are shown in Figure 11. 

From Figure 11, the first 60 corn seed samples had a higher percentage of category 1 with a threshold of 0.55, and all of the category 1 corn seeds were classified correctly and no other category corn seeds were classified to category 1 in this situation. The middle 60 corn seed samples had a larger percentage of category 2 with a threshold of 0.239, and the number 121, 122 and 155 corn seed samples were misclassified as category 2. The last 60 corn seed samples had a larger percentage of category 3 with a threshold of 0.45, and the number 113 corn seed sample was misclassified as category 3.

Among numbers 113, 121, 122 and 155, the number 122 was classified as categories 2 and 3, shown in Table 3. Similar to the above study, the percentages of two categories were compared, and the bigger one was the final category and the final category was shown with the smallest value in the deep black color. In the end, number 122 was classified correctly.

**Figure 12:**
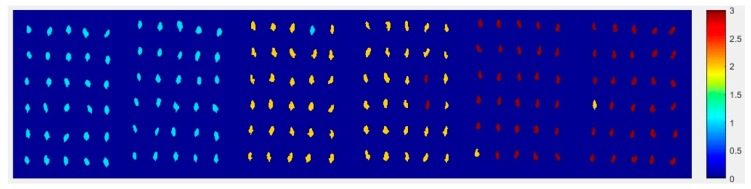
The final visualization images of Haihe78 with the SPA and PLS-DA model with method M2P.

It was also found that the category 2 percentage of numbers 83, 84, 98, 101 112, 113 and 114 corn seeds were lower than 0.239, and they were not included in category 2. The category 3 percentage of numbers 121 and 155 corn seeds were lower than 0.45, and they were not included in category 3. Numbers 113, 121 and 155 corn seeds were classified, but the 83, 84, 98, 101, 112 and 114 corn seeds did not have their own category, so the percentage of each category were compared and shown in Table 4. After subtracting the percentage from the threshold, the final category was shown with the smallest value in the deep black color. At last, the numbers 84, 98, 101, 112 corn seeds were classified as category 2 correctly, while number 83 (should be category 2) was classified as category 1 and number 114 (should be category 2) was classified as category 3. The final classification results for method M2P are shown in Figure 12.

To compare the above results, the number of misclassified corn seed samples are counted. There are six corn seed samples misclassified by method M2M while five corn seed samples are misclassified by method M2P. In some respects, it can be drawn that the effect of method M2P is similar to that of method M2M.

#### 2.4.3. The Visualization Images of Jindan10 

**Figure 13:**
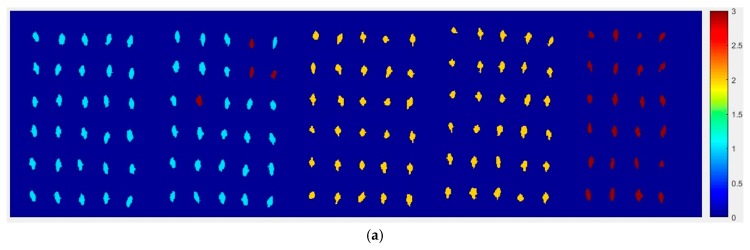

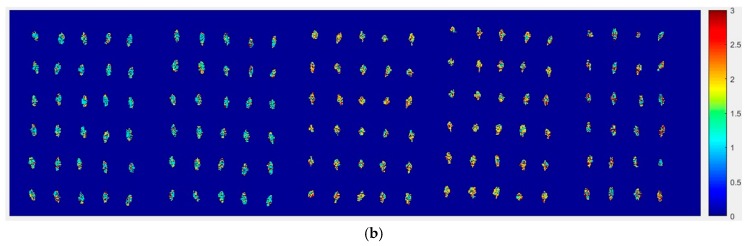
The visualization images (**a**) method M2M and (**b**) method M2P for Jindan10 with the SPA and PLS-DA model.

From Figure 13, the top visualization image (Figure 13a) with the SPA and PLS-DA model was almost matched with the above figure results. The 4 misclassified corn seed samples of Jindan10 was less than that the 17 of Haoyu21 and the 6 of Haihe78. 

**Figure 14:**
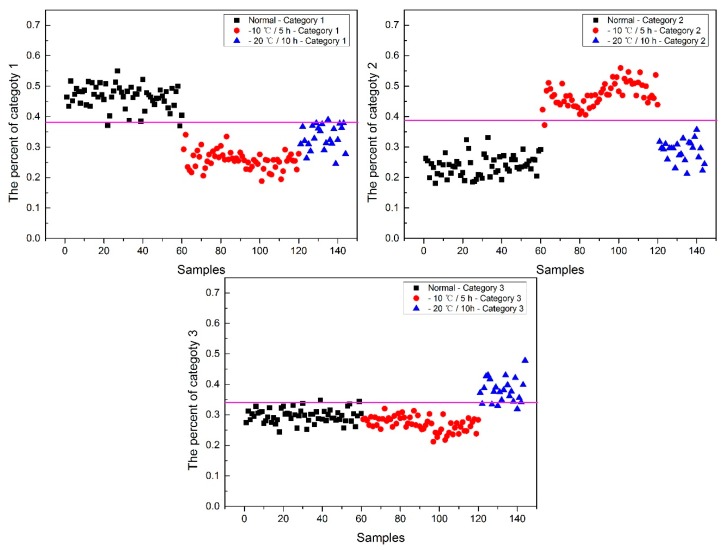
The percentage of each category of Jindan10 with the SPA and PLS-DA model.

Next, the percentage of each category of each corn seed was calculated, and the results are shown in Figure 14.

From Figure 14, the first 60 corn seed samples had a larger percentage of category 1 with a threshold of 0.38, and the number 135 corn seed sample was misclassified as category 1. The middle 60 corn seed samples had a larger percentage of category 2 with a threshold of 0.385, no other category corn seeds were classified as category 2 in this situation. The last 24 corn seed samples had a larger percentage of category 3 with a threshold of 0.34, and the number 39 and 59 corn seed samples were misclassified as category 3.

Among numbers 39, 59 and 135, numbers 39 and 135 were classified to two categories (number 39 was classified to categories 1 and 3, and number 135 was classified to categories 1 and 3), shown in Table 5. Similar to the above study, the percentage of two categories was compared, the bigger one was the final category and the final category was shown with the smallest value in the deep black color. In the end, both numbers 39 and 135 were classified correctly.

**Figure 15:**
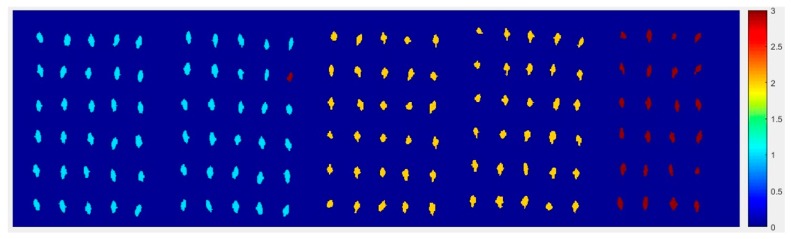
The final visualization images of Jindan10 with the SPA and PLS-DA model with method M2P.

It was also found that the category 1 percentage of numbers 22 and 59 corn seeds was lower than 0.38, and they were not included in category 1. The category 2 percentage of number 62 was lower than 0.385; the category 3 percentage of numbers 122, 127, 130 and 140 corn seeds were lower than 0.34, and they were not included in category 3. The number 59 corn seed was classified from the above study, but the 22, 62, 122, 127, 130 and 140 corn seeds did not have their own category. The percentage of each category was compared and shown in Table 6. After subtracting the percentage from the threshold, the final category was obtained with the smallest value in the deep black color. At last, the number 22, 62, 122, 127, 130 and 140 corn seeds were classified correctly and the final classification results of method M2P are shown in Figure 15.

To compare the above results, the number of misclassified corn seed samples were counted. There were four corn seed samples misclassified by method M2M while one corn seed sample was misclassified by method M2P. Meanwhile, none of the severely freeze-damaged samples were misclassified by method M2P. In some respects, it could be drawn that method M2P had better results than method M2M.

To summarize, the visualization results of three corn varieties using method M2M and method M2P were compared. By setting several category thresholds, and comparing the percentage value or subtracting the percentage from the threshold, method M2P could get fewer numbers of misclassified corn seed samples than in method M2M. In some respects, the method M2P had better results than the method M2M.

## 3. Materials and Methods

### 3.1. Sample Preparation

Three fresh corn seed varieties (Haoyu21, Haihe78 and Jindan10) were collected from the Jiuquan Julong Tengfei Seed Industry Co., Ltd. in Gansu province, China, with a moisture content of about 30% before harvesting in 2017. In order to obtain different freezing-damage corn seeds, the seeds were dried in an oven until the moisture content was 18% and were put in plastic bags for later experiment. Then the seeds were placed in different freezing temperatures for different duration and eventually were divided into three categories (normal samples, slightly freeze-damaged samples, severely freeze-damaged samples). The frozen environment is shown in Table 7. 

180 corn samples (60 normal samples, 60 slightly freeze-damaged samples, 60 severely freeze-damaged samples) of Haoyu21; 180 corn samples (60 normal samples, 60 slightly freeze-damaged samples, 60 severely freeze-damaged samples) of Haihe78; and 144 corn samples (60 normal samples, 60 slightly freeze-damaged samples, 24 severely freeze-damaged samples) of Jindan10. The three varieties of corn samples were randomly divided into calibration sets and validation sets (calibration sets:validation sets = 2:1). Next, the seeds were stored in a refrigerator with a temperature of 4 °C to prevent moisture absorption. Before collecting the hyperspectral images, the moisture content was tested again to ensure all the samples had nearly the same moisture content. The final moisture content was controlled at 13%.

### 3.2. Conductivity Test

Before the collection of hyperspectral images, an electrical conductivity test of corn seeds was conducted to check for the effects of the freeze damage on the corn seeds. Fifty corn seed samples for each treatment condition were weighed and placed in a 500 mL beaker containing 250 mL of deionized water. The conductivity was measured after the corn seed soaked for 8, 12, 16, 20 and 24 h.

### 3.3. Hyperspectral Image Acquisition

#### 3.3.1. Hyperspectral Imaging System

The hyperspectral data of the corn seeds were collected using a VIS/NIR hyperspectral imaging system (Dualix Spectral Imaging, Inc., Sichuan, China). The system consisted of a CCD camera (C8484-05G, Hamamatsu Photonics, Shizuoka, Japan), an imaging spectrometer (Impressor V10E-QE, Spectral Imaging Ltd., Oulu, Finland), a lens (V23-f/2.4 030603, Specim Ltd., Oulu, Finland), line light sources (P/N9130, Illumination Technologies, Inc., East Syracuse, NY, USA) and its controller (2900ER, Illumination Technologies, Inc., East Syracuse, NY, USA), sample stage (GZ02DS20, Guangzheng Instruments Co., Ltd., Beijing, China) and a moving stage controller (PSA200-11-X, Zolix Instruments Co., Ltd., Beijing, China).

The CCD camera, VIS/NIR imaging spectrometer, lens, line light source, sample stage, moving stage and 0.5 mm extension tube were all placed in a dark box. The moving stage was installed in the bottom of the dark box and connected with the moving stage controller. The sample stage was installed on the top of the moving stage and the corn seed was placed on the sample stage; above the sample stage, two line tungsten halogen light sources were installed on both side walls of the dark box, which provided illumination for the corn seeds. The two line light sources were symmetrically at 60º and installed at a height of 26 cm above the moving stage to provide a stable and uniform diffuse reflection light for the corn seeds. The CCD camera, VIS/NIR imaging spectrometer and lens were installed on the top surface of the dark box and were connected to each other vertically. Above the lens, there was a 0.5 mm extension tube between the lens and the VIS/NIR infrared spectrometer. After adding the extension tube, more clear corn seed hyperspectral images were obtained.

#### 3.3.2. Hyperspectral Image Acquisition and Correction

The hyperspectral image acquisition software, Spectracube 2.75b (Spectral Imaging Ltd., Oulu, Finland), was used for image acquisition and correction. At last, the corn seed embryo was placed upside down on black sample stage with an object distance of 28.5 cm, with an exposure time of 1 ms and the moving stage speed of 2.6 mm/s. The hyperspectral images were acquired with a spectral range of 400–1000 nm which had a total of 477 wavelengths. 

In order to overcome the inhomogeneity of the light source intensity at each wavelength and the influence of the dark current of the acquisition sensor, the collected hyperspectral images required black and white correction according to Equation (1).

*I_c_* = (*I_0_* − *I_b_*)/(*I_w_* − *I_b_*) (1)

where *I_c_* is the relative reflectance intensity of each wavelength, *I_0_* is the original reflectance intensity of the hyperspectral image, *I_b_* is the intensity of the dark current, which was obtained by turning off the light source and completely covering the lens with its cap, and *I_w_* is the reflectance intensity of the Teflon white surface (Spectralon, Labsphere Inc., North Sutton, NH, USA), which was obtained under the same conditions as the raw image.

The hyperspectral images were analyzed using the ENVI 4.6.1 software (ITT Visual Information Solutions, Boulder, CO, USA) and MATLAB R2017b (MathWorks, Natick, MA, USA).

### 3.4. Data Analysis

#### 3.4.1. Image Segmentation and Processing

After the hyperspectral images were obtained, the feature image, i.e., the embryo image was extracted for later processing. At first, the average spectrums of 10 pixel × 10 pixel regions of interest (ROI) in the endosperm and embryo were compared, and the gray image in the wavelength of 500 nm was selected as a segment image to separate the embryo (the brighter region) from the whole corn seed (in this method, image enhancement and the Otsu threshold segmentation method was used). Finally, the final embryo-binary image was obtained.

#### 3.4.2. The Spectral Pretreatment Methods

After the embryo-binary image was obtained, it was masked with the original hyperspectral image and the average spectrum of corn seed embryo hyperspectral image was pretreated for spectral feature extraction. Since the spectrometer had low response and noise at the edge of the spectral region, the middle 31 to 460 wavelength spectrum was selected for analysis.

In this paper, multiplicative scatter correction (MSC), standard normal variation (SNV) and 5 points and 3 times smoothing (5-3 smoothing) pretreatment methods were applied [43,44].

The MSC method is used to correct the scattering of each sample’s spectrum, get the desired spectrum, and remove the undesirable scatter effect which can improve the signal-to-noise ratio [44].

The SNV method is commonly used to eliminate spectral errors among samples due to different solid particle sizes, scattering, or measurement path lengths. It converts the data mean to 0 and the standard deviation to 1 and is generally used for scatter correction and to remove the slope variation from the spectra [43]. 

The smoothing method can effectively remove high-frequency noise which may be produced by instrument noise, random errors, etc., which can keep the original useful signal information and improve the signal-to-noise ratio at the same time [44]. In this study, 5-3 smoothing was applied which is based on 5 points and 3 times polynomial fitting, and the smoothing time was 2000.

#### 3.4.3. Spectral Feature Extraction

It is well known that a hyperspectral image has a large amount of spectral data and the whole spectral wavelength contains much noise and irrelevant information. According to the introduction, it is necessary to apply some algorithms (in this study, the successive projection algorithm (SPA), principal component analysis (PCA) and other methods were applied) which have faster speed in data computation and higher accuracy to obtain important wavelengths and establish the classification model [42].

SPA is a forward selection algorithm that looks for variable combinations with minimal redundancy information in the variable matrix to minimize collinearity among the variables. Therefore, the SPA is used to extract the characteristic wavelengths during spectral data analysis processing [42].

PCA is a commonly used dimension reduction mapping method, which maps original features with strong original correlation into a set of new features. Each new feature constructed is a linear combination of the original features. Each new feature is not related to each other [45].

X-loading method uses spectral variables for partial least-squares modeling, and the wavelengths are extracted based on the absolute value of the regression coefficients for each wavelength. As the absolute value of the regression coefficient gets larger, the greater the influence of these wavelengths on the modeling, that is, the final extracted feature wavelength [42].

#### 3.4.4. The Classification Methods

In this paper, the partial least squares-discriminant analysis (PLS-DA), K-nearest neighbor (KNN) and support vector machine (SVM) models were established for classification of different frozen corn seeds.

The PLS-DA is based on the partial least squares (PLS) technique that is a commonly used in multivariate statistical analysis methods [46]. The main principle of the PLS-DA model is briefly described as Equation (2):

Y = X_n*p_ B + E (2)

where Y is the matrix of the response variables that relates to the measured sample categories; X is the n*p matrix of the spectral variables for each measured sample category; n is the number of samples, p is the number of variables; B is the matrix of regression coefficients for the spectral variables and E is the matrix of residuals. The number of main components is optimized by using ten-fold cross validation during the model development and updating stages.

KNN classifies by measuring the distance (the distance can be Euclidean distance or Manhattan distance) between different feature values [47]. If a sample is in the feature space, most of the k most similar samples belong to a certain category, and the sample also belongs to this category. The main principles of KNN are as follows:

(1) Calculating the distance d (this paper Euclidean distance was applied as d) between the test data and each set of training data; (2) Sorting the distance increasingly; (3) Selecting the K points with the smallest distance; (4) Calculating the frequency f of each category of the K points; and (5) The category with the highest frequency among the K points is the predictive category for the test data. K is optimized by using ten-fold cross validation during the model development and updating stages.

SVM is an important classification method and has many unique advantages in solving small sample sets, nonlinear and high-dimensional pattern recognition problems [48]. It establishes the model from the limited training samples and obtains small errors for the independent test set. It tries to improve the generalization ability of the learning machine. The data input space is mapped into a high-dimensional feature space through a kernel function (in this paper, the radial basis function (RBF) kernel function was applied); and c (the penalty factor) and g (the radial width of the kernel function) are the two main parameters of the SVM method, which are optimized using a grid-search algorithm coupled with ten-fold cross validation during the model development and updating stages.

To analyze the classification accuracy results, there were three aspects can be considered: (1) the results of different pretreatment methods at full-band treatment; (2) the results of different wavelength selection algorithms with the same pretreatment method; and (3) the results of different classification methods with the same pretreatment method and the same wavelength selection algorithm. The >80% classification accuracy results of both the calibration sets and the validation sets could be observed clearly by a pink 80% line among the three corn varieties.

#### 3.4.5. The Visualization Images of the Classification Results

When the PLS-DA, KNN, and SVM methods were applied to establish the classification models, the robust classification model was selected to achieve the visualization of classification results of the corn seeds. 

The average spectrum and the pixel spectrum are explained. When the ROI of a hyperspectral image is extracted, it contains many pixels, and each pixel corresponds to a spectrum; in this paper, the average spectrum refers to the average spectrum of the seed embryo ROI, the pixel spectrum refers to the spectrum of each pixel in the seed embryo ROI. 

In this paper, the visualization images were obtained in Method M2M and Method M2P. Method M2M: the classification results were obtained from the above study and each corn seed was corresponding with the classification value. Method M2P: the spectrum of each pixel of the embryo images was classified, and each pixel of the corn seed was corresponding with the classification value. Mean-spectrum to mean-spectrum (M2M) means that both of the calibration and validation sets were the mean spectrums of the ROI; Mean-spectrum to pixel-spectrum (M2P) means that the calibration sets were the mean spectrums of the ROI while the validation sets were the spectrum of each pixel in the seed embryo ROI.

It is known that the surface of the corn seed sample is not very flat where the height of the embryo edge is higher than the height of the embryo inside, thus, in this paper, 1~3 thresholds for each category were set to classify the different degrees of freeze-damage in corn seed after the percentage of each category was calculated.

## 4. Conclusions

Conductivity test is a general method to distinguish different degrees of freeze-damage in corn seed. The more serious the freeze damage is, the higher its conductivity. In this feasibility attempt to classify different degrees of freeze-damage in corn seed with hyperspectral imaging technology, four different pretreatment methods (no pretreatment, SNV, MSC and 5-3 smoothing), four wavelength selection algorithms (SPA, PCA, X-loading and full-band methods) and three different classification modeling methods (PLS-DA, KNN and SVM) were applied to find a relatively better method for the three different corn seed varieties. In order to better represent the freeze damage of the seed embryos, comparisons were made between method M2M and method M2P.

The following conclusions are drawn from this study:

(1) By using related image preprocessing methods on the gray image of corn seeds at 500 nm wavelength, the final embryo hyperspectral images can be clearly obtained to achieve the following classification of different degrees of freeze-damaged corn seed.
(2) The 5-3 smoothing pretreatment method has higher classification accuracy than the other pretreatment methods from the results of different pretreatment methods at full-band treatment. The classification accuracy almost reaches 90%, maybe because the pretreatment has the advantages of keeping the signal from the original spectrums and improving the signal-to-noise ratio.
(3) The classification accuracies of the full-band, SPA, PCA and X-loading algorithms can be sorted as follows: the classification accuracies of the full-band method are higher than those of the SPA and PCA methods, and the X-loading method has the lowest classification accuracy from the results of different wavelength selection algorithms with the same preprocessing method. Maybe this is because a great deal of information about the corn seeds can be got in the full band situation, but in some way, it necessary to find several wavelengths to reduce the modeling time and improve the efficiency, so the SPA algorithm could be a good choice.
(4) The classification accuracies of the PLS-DA, KNN and SVM methods can be sorted as follows: the PLS-DA modeling method has the best classification accuracy compared to the SVM and KNN modeling methods, while the KNN modeling method has the lowest classification accuracy.
(5) By setting several category thresholds, and comparing the percentage value or subtracting with method M2P, fewer numbers of misclassified corn seed samples can be obtained. In some respects, method M2P has better results than method M2M.

Based on the above several conclusions, it is feasible that the hyperspectral imaging technology used to establish classification models for the embryos of corn seeds with different degrees of freeze damage. The smoothing method and wavelength selection method can be applied to modeling to improve the signal-to-noise ratio, classification efficiency and result accuracy of the model, although good modeling results can be obtained in the full-band case. The method M2P allowed visualization of the classification result of each embryo pixel and the final classification result is better than the method M2M.

## Figures and Tables

**Table 1:** The percentages of the two categories of Haoyu21 corn seed samples.

The Number (the Original Category) of the Sample	The Percentage—Threshold of Category 1	The Percentage—Threshold of Category 2	The Percentage—Threshold of Category 3
83 (2)	**0.38833–0.37**	0.32435–0.32	0.28732–0.44
167 (3)	0.17004–0.37	0.32591–0.32	**0.50405–0.44**

**Table 2:** The percentage and subtraction of each uncertain Haoyu21 corn seed sample.

The Number (the Original Category) of the Sample	The Percentage—Threshold (Subtraction) of Category 1	The Percentage—Threshold (Subtraction) of Category 2	The Percentage—Threshold (Subtraction) of Category 3
51 (1)	0.31313–0.37 (0.0587)	**0.28535–0.32 (0.03465)**	0.40152–0.44 (0.03848)
86 (2)	0.321–0.37 (0.049)	**0.31026–0.32 (0.00974)**	0.36874–0.44 (0.07126)
115 (2)	**0.36018–0.37 (0.00982)**	0.28308–0.32 (0.03692)	0.35673–0.44 (0.08327)
116 (2)	**0.36364–0.37 (0.00636)**	0.26477–0.32 (0.05523)	0.37159–0.44 (0.06841)

**Table 3:** The percentage of the two categories of Haihe78 corn seed samples.

The Number (the Original Category) of The Sample	The Percentage—Threshold of Category 1	The Percentage—Threshold of Category 2	The Percentage—Threshold of Category 3
122 (3)	0.22491–0.55	0.26965–0.239	**0.50544–0.45**

**Table 4:** The percentage and subtraction of each uncertain Haihe78 corn seed sample.

The Number (the Original Category) of the Sample	The Percentage—Threshold (Subtraction) of Category 1	The Percentage—Threshold (Subtraction) of Category 2	The Percentage—Threshold (Subtraction) of Category 3
83 (2)	**0.39402–0.55 (0.15598)**	0.19421–0.239 (0.04479)	0.41176–0.45 (0.03824)
84 (2)	0.39179–0.55 (0.15821)	**0.22754–0.239 (0.01146)**	0.38067–0.45 (0.06933)
98 (2)	0.38179–0.55 (0.16821)	**0.23726–0.239 (0.00174)**	0.38095–0.45 (0.06905)
101 (2)	0.34878–0.55 (0.20122)	**0.23059–0.239 (0.00841)**	0.42063–0.45 (0.02937)
112 (2)	0.47799–0.55 (0.07201)	**0.19227–0.239 (0.04673)**	0.32974–0.45 (0.12026)
114 (2)	0.34722–0.55 (0.20278)	0.20313–0.239 (0.03587)	**0.44965–0.45 (0.00035)**

**Table 5:** The percentage of the two categories of Jindan10 corn seed samples.

The Number (the Original Category) of the Sample	The Percentage—Threshold of Category 1	The Percentage—Threshold of Category 2	The Percentage—Threshold of Category 3
39 (1)	**0.38468–0.38**	0.26813–0.385	0.34719–0.34
135 (3)	0.38983–0.38	0.21243–0.385	**0.39774–0.34**

**Table 6:** The percentage and subtraction of each uncertain Jindan10 corn seed sample.

The Number (the Original Category) of the Sample	The Percentage—Threshold (Subtraction) of Category 1	The Percentage—Threshold (Subtraction) of Category 2	The Percentage—Threshold (Subtraction) of Category 3
22 (1)	**0.37094–0.38 (0.00906)**	0.32401–0.385 (0.06099)	0.30505–0.34 (0.03495)
62 (2)	0.34061–0.38 (0.03939)	**0.37212–0.385 (0.01288)**	0.28727–0.34 (0.05273)
122 (3)	0.36697–0.38 (0.01303)	0.29702–0.385 (0.08798)	**0.33601–0.34 (0.00399)**
127 (3)	0.36983–0.38 (0.01017)	0.29603–0.385 (0.08897)	**0.33414–0.34 (0.00586)**
130 (3)	0.36148–0.38 (0.01852)	0.30871–0.385 (0.07629)	**0.32982–0.34 (0.01018)**
140 (3)	0.324–0.38 (0.056)	0.35688–0.385 (0.02812)	**0.31912–0.34 (0.02088)**

**Table 7:** The frozen condition (freezing temperature and freezing duration) of corn seeds.

Frozen Condition	Normal	Slight Freeze-Damage	Severely Freeze-Damage
Freezing temperature	Room temperature	−10 °C	−20 °C
Freezing duration	/	5 h	10 h

## Appendix A

**Table A1:** The selected wavelengths using different methods under different pretreatments of Haoyu21.

**No Pretreatment**	**The Number of Wavelengths**	**Wavelength/nm**
SPA	5	445.44 484.16 645.23 977.66 984.09
PCA	6	465.96 482.94 535.58 608.84 667.92 878.6
X-loading	7	461.12 637.68 861.89 881.17 918.48 968.65 976.37
Full-band	430	444.24~985.27
**MSC**	**The number of wavelengths**	**Wavelength/nm**
SPA	5	470.8 474.44 492.68 824.66 906.9
PCA	11	459.91 465.96 469.59 470.80 535.58 684.36 832.3500 847.7600 878.6 919.76 968.65
X-loading	7	472.01 854.1 863.18 865.75 964.79 976.37 977.66
Full-band	430	444.24~985.27
**SNV**	**The number of wavelengths**	**Wavelength/nm**
SPA	6	449.05 455.08 470.8 784.94 906.9 927.48
PCA	11	459.91 465.96 469.59 470.8 535.58 684.36 832.35 847.76 878.6 919.76 968.65
X-loading	7	472.01 854.18 863.18 865.75 964.79 968.65 977.66
Full-band	430	444.24~985.27
**5-3 smoothing**	**The number of wavelengths**	**Wavelength/nm**
SPA	8	486.59 511.01 530.66 549.16 656.57 689.42 887.6 904.33
PCA	9	449.05 467.17 484.16 641.46 671.71 861.89 874.74 913.33 981.51
X-loading	7	449.05 451.46 463.54 467.17 647.75 864.46 976.37
Full-band	430	444.24~985.27

**Table A2:** The selected wavelengths using different methods under different pretreatments of Haihe78.

**No Pretreatment**	**The Number of Wavelengths**	**Wavelength/nm**
SPA	3	465.96 523.28 637.68
PCA	10	451.46 458.7 478.08 540.51 551.63 605.09 756.83 864.46 870.89 876.03
X-loading	7	464.75 478.08 864.46 868.32 870.89 891.46 969.94
Full-band	430	444.24~985.27
**MSC**	**The number of wavelengths**	**Wavelength/nm**
SPA	6	461.12 533.12 735.15 840.06 843.91 921.05
PCA	14	457.49 462.33 464.75 482.94 509.79 525.74 531.89 643.97 756.83 814.4 842.62 851.61 877.32 924.91
X-loading	6	457.49 531.89 863.18 869.6 969.94 976.37
Full-band	430	444.24~985.27
**SNV**	**The number of wavelengths**	**Wavelength/nm**
SPA	6	465.96 508.56 557.82 636.43 763.21 864.46 919.76 945.5 960.94
PCA	12	457.49 464.75 482.94 509.79 525.74 531.89 643.97 756.83 814.4 842.62 851.61 924.91
X-loading	6	457.49 484.16 863.18 869.6 969.94 976.37
Full-band	430	444.24~985.27
**5-3 smoothing**	**The number of wavelengths**	**Wavelength/nm**
SPA	9	465.96 508.56 557.82 636.43 763.21 864.46 919.76 945.5 960.94
PCA	10	456.29 472.01 540.51 602.59 662.88 781.1 870.89 912.04 966.08 972.51
X-loading	7	449.05 462.33 475.65 865.75 969.94 971.23 976.37
Full-band	430	444.24~985.27

**Table A3:** The selected wavelengths using different methods under different pretreatments of Jindan10.

**No Pretreatment**	**The Number of Wavelengths**	**Wavelength/nm**
SPA	9	450.26 451.46 452.67 465.96 491.46 520.82 964.79 978.94 985.37
PCA	8	449.05 484.16 490.24 525.74 554.1 665.4 868.32 919.76
X-loading	7	450.26 465.96 491.46 877.32 964.79 967.37 976.37
Full-band	430	444.24~985.27
**MSC**	**The number of wavelengths**	**Wavelength/nm**
SPA	6	472.01 551.63 722.43 904.33 915.9 985.37
PCA	16	462.33 472.01 479.3 498.78 506.11 523.28 539.28 550.39 655.31 833.64 842.62 845.19 859.32 883.75 888.89 919.76
X-loading	7	457.49 459.91 463.54 484.16 865.75 879.89 984.09
Full-band	430	444.24~985.27
**SNV**	**The number of wavelengths**	**Wavelength/nm**
SPA	9	472.01 551.63 722.43 904.33 915.9 922.34 923.62 951.93 985.37
PCA	18	456.29 462.33 472.01 479.3 484.16 498.78 506.11 523.28 550.39 655.31 758.1 833.64 842.62 845.19 859.32 883.75 888.89 919.76
X-loading	7	457.49 459.91 463.54 484.16 865.75 873.46 984.09
Full-band	430	444.24~985.27
**5-3 smoothing**	**The number of wavelengths**	**Wavelength/nm**
SPA	14	462.33 473.23 485.37 503.67 526.97 571.46 632.66 665.4 804.14 864.46 886.32 921.05 949.36 963.51
PCA	13	446.64 450.26 469.59 491.46 523.28 526.97 554.1 661.61 872.17 918.48 939.06 962.22 980.23
X-loading	7	450.26 485.37 878.6 922.34 960.94 968.65 977.66
Full-band	430	444.24~985.27
